# Measurement and evaluation of specific absorption rate and temperature elevation caused by an artificial hip joint during MRI scanning

**DOI:** 10.1038/s41598-020-80828-7

**Published:** 2021-01-13

**Authors:** Youngseob Seo, Zhiyue J. Wang

**Affiliations:** 1 Division of Chemical and Biological Metrology, Korea Research Institute of Standards and Science, 267 Gajeong-ro, Doryong-dong, Yuseong-gu, Daejeon, 34113 Republic of Korea; 2 Department of Radiology, University of Texas Southwestern Medical Center, Dallas, TX USA; 3 Department of Radiology, Children’s Health, Dallas, TX USA

**Keywords:** Medical research, Engineering

## Abstract

A primary safety concern in a magnetic resonance imaging environment is heating of metallic implants by absorbing radiofrequency (RF) energy during MRI scanning. Experimental measurement in conjunction with computational modeling was used to evaluate the risk of biological tissue injury from the RF heating of artificial hip joints by obtaining both specific absorption rate (SAR) and temperature elevation at 1.5 T and 3 T MRI systems. Simulation result showed that high SAR and high temperature appeared near both head and tail sections of the artificial hip joints. For five different 1.5 T and 3 T MRI systems, measured temperature location showed that high temperature rises occurred near both head and tail regions of the metallic hip joints. Measured SAR value of 24.6 W/kg and the high temperature rise (= 4.22 °C) occurred in the tail region of the hip joint at 1.5 T, which was higher than the limits for temperature required by the international electrotechnical commission 60601-2-33. We have demonstrated the feasibility of evaluating RF heating of metallic hip joints during MRI scans.

## Introduction

Magnetic resonance imaging (MRI) plays an important role in medical diagnoses of diseases. High-field MRI has rapidly gained acceptance in the MR community for both clinical applications and researches in the past decades. The most important advantage of high-field MRI is the increase in SNR leading to decreased acquisition time, increased image resolution or a combination of both. MR safety is becoming an important issue as the clinical use of high-field MRI scanners increases. A primary safety concern during MRI scanning is metallic medical implant heating by absorbing radiofrequency (RF) energy^1,2^. This risk depends on RF power, static magnetic field strength, the shape and location of the implant, pulse sequences and imaging parameters^3–5^.

Therefore, it is important to measure the specific absorption rate (SAR) of the RF energy absorbed by the body accurately. The international electrotechnical commission (IEC) standard requires that the average SAR values are restricted to 4 W/kg for the body and to 3.2 W/kg for the head for 6 min duration^6^. According to the U.S. Food and Drug Administration, the average SAR values are limited to 3 W/kg when the head is scanned for 10 min and to 4 W/kg when the body is scanned for 15 min^7^. In addition, the local SAR levels averaged over 10 g of tissue also need to be restricted even though a higher level is allowed. The American Society for Testing and Materials (ASTM) established technical standards for assessing RF heating caused by passive implantable medical devices during MRI scans^8^.

Millions of total joint replacement are performed annually worldwide, and the number is gradually increasing every year^9^. Total hip replacement was initially largely restricted to either elderly people or individuals with locomotor limitations associated with other comorbidities. Today, however, patients seek so-called high performance hips to meet their expectations and aspirations. The replacement of the damaged hip joint by an artificial device is a surgical procedure widely performed in orthopedics in the last decades^10^. The risk of hyperthermic tissue damage is relatively serious for patients who have insensate limbs and those who are under anesthesia during MRI scans.

Commercial MRI scanners provide an estimated SAR level for each scan under an average condition without any implant; this level is calculated from the RF waveforms and sequence parameters, system calibration, coil factors, patient weight and height, etc. It has been known that SAR values indicated by clinical MRI scanners may not be reliable^11–15^, and much higher local SAR induced by the presence of a metal implant can cause damages to the tissue. Numerical simulations of RF energy deposition have been performed to predict SAR levels in anatomical models consisting of homogeneous cylinders, spheres, or heads^16–23^. There is a large range of variability in SAR levels for different pulse sequences. In nearly all cases, radiologists, physicists, and technologists do not have a tool with which to verify the power level independently. Direct estimation of SAR values independent of the level calculated by MRI scanners is therefore desirable.

In this study, we aimed to measure SAR levels of metallic hip implants and evaluate the risk of injury from RF-induced heating of the hip implants from different vendors during high-field MRI scanning. Computer simulation was performed for one case to examine the spatial distribution of the electric field (E-field) which causes heating.

## Results

### Numerical simulation of SAR and RF-induced heating

Supplementary Fig. 1 shows a transverse slice of the RF magnetic field (B1^+^) distributions obtained in the center of the unloaded body coil in the empty condition after the coil tuning process. The result indicates that the B1^+^ field was uniform in the empty birdcage coil except in regions very close to the birdcage coil.

**Figure 1:**
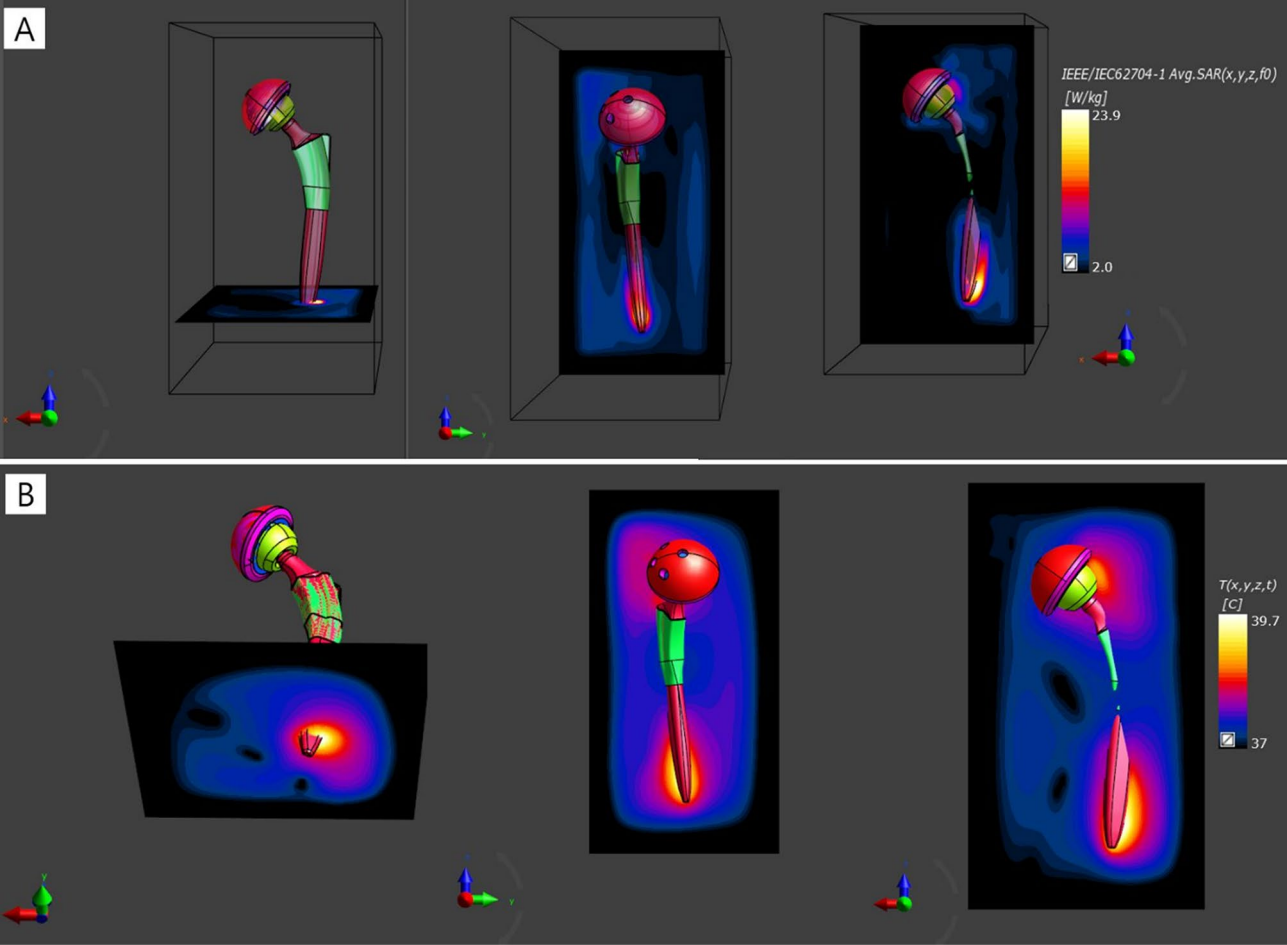
SAR (**A**) and Temperature distribution (**B**) near an artificial hip joint using Sim4Life version 4.4 software (Zurich Med Tech, Zurich, Switzerland. https://zmt.swiss/sim4life/). The local SAR values were normalized to the whole-body averaged SAR of 2 W/kg. The SAR value was 23.9 W/kg near the tail of the artificial hip joint. The maximum temperature (= 39.7 °C at 8 min exposure at MRI, compared to base temperature at 37 °C) appear near the tail of the artificial hip joint.

Figure 1 shows computer-simulated SAR distribution and temperature rise near artificial hip joints. Both high SAR and high temperature appear near both head and tail sections of the artificial hip joints. In particular, SAR value was 23.9 W/kg with background SAR (= 2.0 W/kg) in the tail of the artificial hip joints.

### Experimental studies

#### Reference local SAR and local SAR levels in the presence of hip joint implant

**Figure 2:**
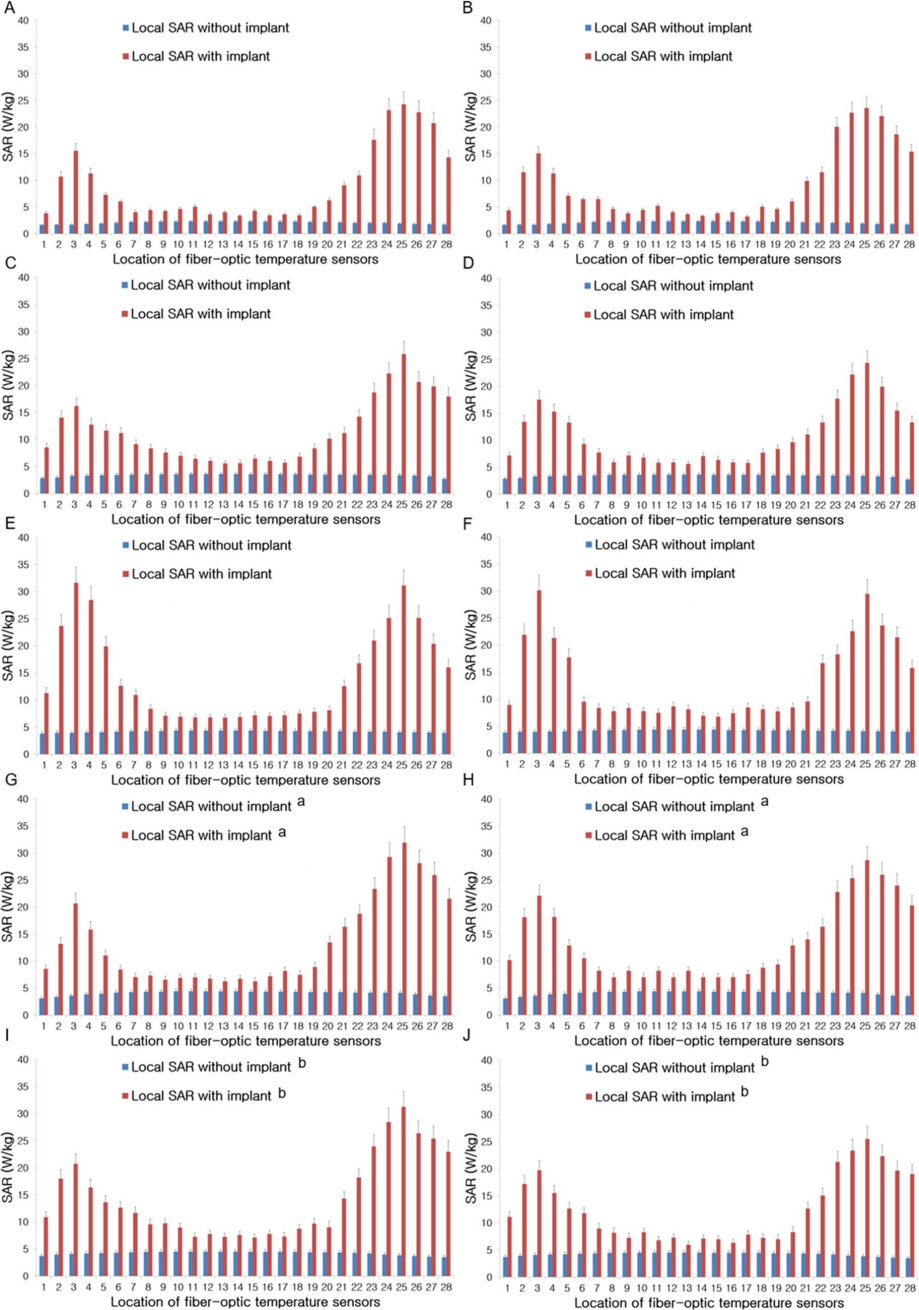
Local SAR levels with and without the implant on the left (**A**) and right (**B**) sides of the metallic hip joints at 1.5 T Philips with T1w TSE sequence, left (**C**) and right (**D**) at 1.5 T GE with IR TSE sequence, left (**E**) and right (**F**) at 3 T Siemens with T1 TIRM sequence and left (**G** and **I**) and right (**H** and **J**) at 3 T Philips with T1w TSE sequence (a, b = two same model Philips 3 T MRI systems) and imaging parameters as described in Supplementary Table 1. High SAR values occurred near the head and tail regions of the hip joint implants as compared with the local SAR without the implant.

Reference local SAR refers to the SAR level when the implant is absent. Figure 2 shows reference local SAR without the implant and SAR levels with the implant at each of fifty-six temperature sensor locations on both sides of the artificial hip joints (28 fiber-optic temperature sensors on each joint) at 1.5 T Philips with T1w TSE sequence, 1.5 T GE with IR TSE sequence, 3 T Siemens with T1 TIRM sequence, and two same model 3 T Philips MRI systems with T1w TSE sequence and imaging parameters as described in Supplementary Table 1.

**Table 1:** Experimentally measured SAR (mean ± SD) using four optical E-field sensors at Philips 1.5 T and GE 1.5 T MRI systems and the average of the local SAR measurements with and without artificial hip joints at all locations from the head to the tail of the implants. The local SAR values were obtained from temperature sensors.

Image sequence	Philips 1.5 T			GE 1.5 T		
	T1w TSE	T1w SPIR	T2w TSE	T1w TSE	IR TSE	T2w TSE
Averaged local SAR with (without) implant (W/kg)	9.37 (2.08)	6.72 (2.08)	4.91 (2.08)	11.83 (3.42)	11.31 (3.42)	11.56 (3.42)
SAR at OEFS #1	15.19 ± 1.22	7.96 ± 0.64	6.15 ± 0.46	10.56 ± 0.84	16.92 ± 1.35	10.88 ± 0.87
SAR at OEFS #2	23.82 ± 2.14	12.84 ± 1.16	9.41 ± 0.78	8.61 ± 0.75	22.13 ± 1.77	9.21 ± 0.79
SAR at OEFS #3	24.6 ± 2.16	11.46 ± 1.03	8.98 ± 0.76	18.44 ± 1.66	24.3 ± 2.12	18.83 ± 1.68
SAR at OEFS #4	16.82 ± 1.35	8.93 ± 0.68	5.67 ± 0.36	14.83 ± 1.18	12.73 ± 1.14	15.77 ± 1.26

OEFS = optical E-field sensors, SD = standard deviation, SPIR = spectral pre-saturation with inversion recovery, TSE = Turbo spin echo.

#### Experimentally measured SAR value at 1.5 T and 3 T MRI scanners

Tables 1 and 2 represent the measured SAR values by the four optical E-field sensors positioned at the head and tail sections of the artificial hip joints where the heating is maximum for each scanner according to the results of the numerical simulation. In addition, Tables 1 and 2 show the average of the local SAR measurements with and without the hip joints at all locations from the head to the tail of the implants.

**Table 2:** Experimentally measured SAR (mean ± SD) using four optical E-field sensors at Siemens 3 T and two of same model Philips 3 T systems the average of the local SAR measurements with and without artificial hip joints at all locations from the head to the tail of the implants.

Image sequence	Siemens 3 T			Philips 3T^a^			Philips 3 Tb		
	T1w TSE	T1 TIRM	T2w TSE	T1w TSE	T1w SPIR	T2w TSE	T1w TSE	T1w SPIR	T2w TSE
Averaged local SAR with (without) implant (W/kg)	12.09 (4.19)	13.92 (4.19)	11.73 (4.19)	13.61 (4.07)	12.41 (4.07)	11.93 (4.07)	13.45 (4.24)	11.94 (4.24)	11.89 (4.24)
SAR at OEFS #1	18.32 ± 1.47	19.14 ± 1.35	16.77 ± 1.34	19.02 ± 1.45	18.51 ± 1.38	23.35 ± 1.78	18.16 ± 1.45	19.37 ± 1.45	16.38 ± 1.21
SAR at OEFS #2	19.86 ± 1.79	19.64 ± 1.47	20.57 ± 1.85	25.03 ± 2.15	18.39 ± 1.56	14.68 ± 1.23	22.3 ± 1.98	16.68 ± 1.41	12.65 ± 1.04
SAR at OEFS #3	21.51 ± 1.84	22.74 ± 2.01	23.46 ± 2.11	28.8 ± 2.39	13.53 ± 1.12	18.93 ± 1.61	27.6 ± 2.28	13.74 ± 1.14	20.3 ± 1.73
SAR at OEFS #4	27.68 ± 2.16	28.4 ± 2.17	25.42 ± 2.03	17.43 ± 1.29	13.18 ± 1.05	15.57 ± 1.15	19.32 ± 1.45	14.35 ± 1.02	13.82 ± 0.99

a, b = two same model Philips 3 T MRI systems, SD = standard deviation, TIRM = turbo inversion recovery magnitude, SPIR = spectral pre-saturation with inversion recovery, TSE = Turbo spin echo.

On the Philips 1.5 T MRI system, the measured maximum SAR value was 24.6 W/kg at OEFS #3 (near tail region of the hip joint) for the T1w TSE sequence. On the GE 1.5 T, the measured maximum SAR value was 24.3 W/kg at OEFS #3 (near tail region) for the IR TSE sequence. On the Siemens 3 T, the measured maximum value was 28.4 W/kg at OEFS #4 (near head area of the hip joint) for T1 TIRM sequence. On the two same model Philips 3 T, the measured maximum values were 28.8 and 27.6 W/kg at OEFS #3 (near tail region) for the T1w TSE sequence, respectively.

#### Comparison of SAR levels obtained from E-field measurement and temperature measurement at the hottest area

The difference between the E-field-based and temperature-based SAR quantification was ≤ 8.8% at 1.5 T Philips, ≤ 7.4% at 1.5 T GE, ≤ 8.3% at 3 T Siemens, ≤ 9.1 and 9.4% at two same model 3 T Philips MRI systems, respectively.

### Measured temperature rise at 1.5 T and 3 T MRI scanners

As a result of demonstrating that heat losses to the environment are minimized during measurement, initial phantom temperatures measured by the temperature sensors ranged from 27.8 to 28.3 °C, compared to temperature ranging from 23.6 to 25.4 °C inside the magnet bores, and it took at least 3 h 52 min to reach thermal equilibrium with the environment on five MRI systems.

**Figure 3:**
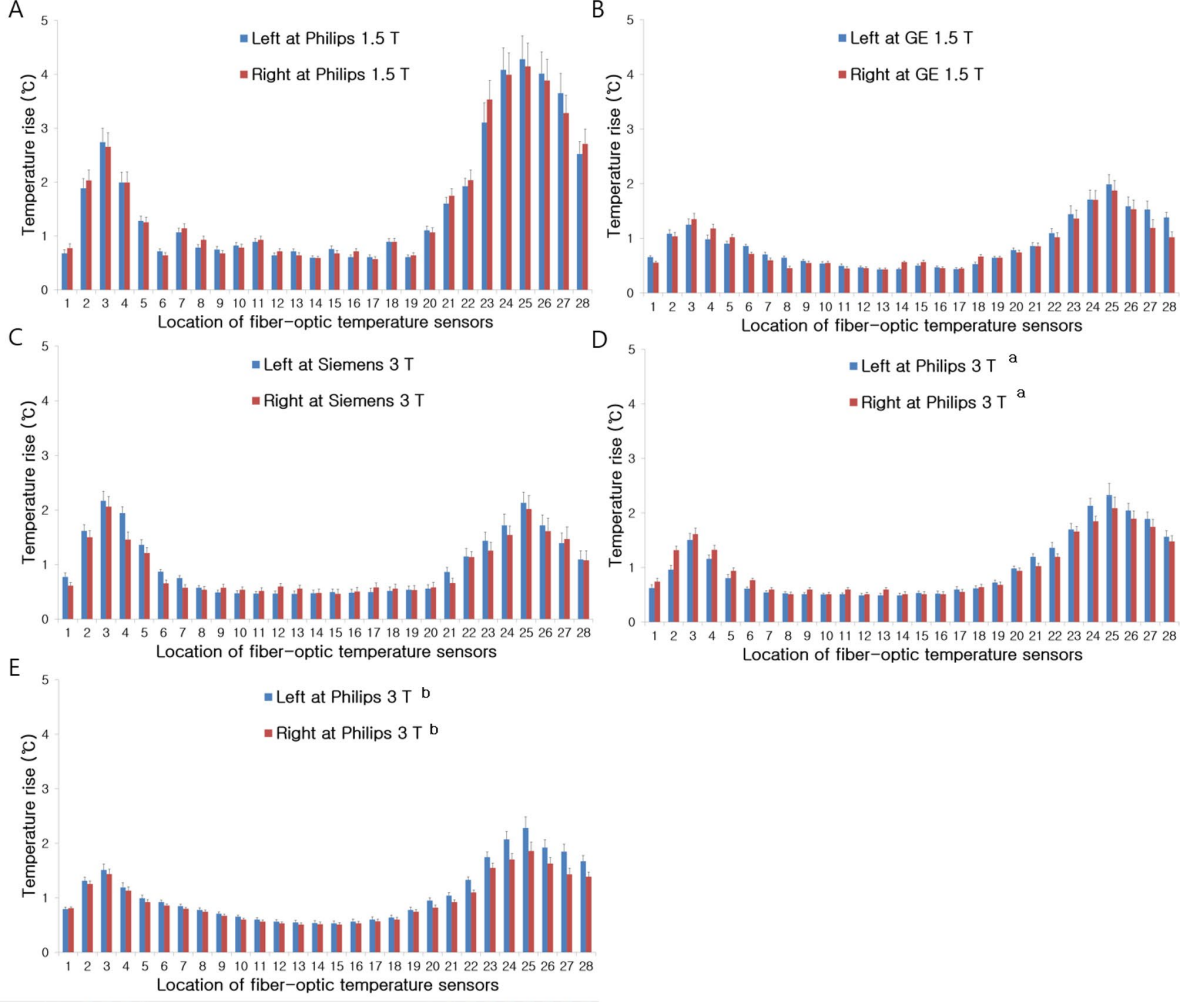
Temperature rise measured via fiber-optic Bragg grating temperature sensors on both sides of the artificial hip joints at 1.5 T Philips with T1w TSE sequence (**A**), 1.5 T GE with IR TSE sequence (**B**), 3 T Siemens with T1 TIRM sequence (**C**), and 3 T Philips with T1w TSE sequence (**D** and **E**) and imaging parameters as described in Supplementary Table 1. Temperature rise by other two sequences for each scanner is not shown here. The column height is the temperature rise and the error bar is the standard deviation. The peak temperature elevation appears near the head or tail of the artificial hip joint. (a, b = two same model Philips 3 T MRI systems).

Supplementary Fig. 2 shows the measured temperature as a function of scan time for three different sequences at one peak temperature location near the tip part of the hip implant. Temporal temperature increased gradually with continuous RF irradiation during MRI scans. Figure 3 shows temperature rise measured via the FBG sensors positioned in the vicinity of a pair of the artificial hip joints for each scanner with the T1w TSE, IR TSE or T1 TIRM sequence and imaging parameters as described in Supplementary Table 1. The measured peak temperature rise was 4.22 °C for the T1w TSE sequence (scan time = 12 min 12 s) at 1.5 T Philips and 1.99 °C for the IR TSE sequence (scan time = 5 min 20 s) at 1.5 T GE near the tail region of the hip joint, respectively. At 3 T Siemens, the peak temperature rise was 2.16 °C near head area of the hip joint for T1 TIRM sequence (scan time = 4 min 44 s). On the two same model Philips 3 T, the peak temperature rises were 2.33 and 2.24 °C near the tail region for the T1w TSE sequence (scan time = 5 min 6 s), respectively.

## Discussion and conclusion

The growing popularity of MR imaging suggests that more patients with implanted metallic devices will undergo MRI scans. The major concern in this regards is the MR safety of patients or health risk posed by RF-induced heating due to hip joint implants, cardiovascular implants, knee prosthetic joints, neurosurgical implants, implantable microstiumulators, orthopedic plate implants, straight stainless steel rods and stents^2–4,24–35^.

A previous phantom study has reported that temperature near metallic hip joint implants rose from 1 to 9 °C at six measured positions from the tip to the head of the hip implants^33^. However, there was a big difference between the simulated and measured temperatures at the tip in the titanium alloy implant using only one pulse sequence at 1.5 T alone. Maximum temperature rises were seen at the tip of the implants and minimum temperature rises at the head of the implants. In addition, for electro-magnetic field analysis, the electric current of RF coils was estimated from the RF power displayed on the MR console and the impedance of each RF coil without direct measurement. Another phantom study reported that temperatures at three locations in titanium hip prostheses were measured experimentally only at 1.5 T with only one MRI sequence and that the maximum temperature rise was approximately 1.5 °C at the tip of the implants^34^. Although both numerical modeling and experimental measurements were performed, the method of SAR and temperature measurements was not specifically described in the experiment and it did not show experimental SAR measurements around the hip joint implants at 1.5 T. In addition, four local temperature values were shown near the tip and neck part of the hip joint implant, excluding the head. The maximum difference between numerical analysis and experiment was 81.3% depending on the measurement locations because the numerical model was simplified and was not an ideal experimental conditions. Another numerical calculation showed that temperature increase was approximately 15 °C at the tip of two knee prosthetic joint implants^35^. However, the results from previous numerical simulation by others cannot be comparable with our results of the hip joint implant because RF-induced heating depends on the implant geometry, orientation and location in the bore, electrical properties of the implant material, phantom design, etc.

RF dosimetry using the E-field probes will be a valuable tool for MR safety. This method does not require long scan time to observe a SAR level. In part of the present study, we performed SAR measurements using optical E-field sensors. The E-field sensors were calibrated with traceability and the results could be read easily. The E-field is generally not uniform even with uniform B1 field. The sensors employed in this study have small sizes and in effect measure the power level in a spatial point. The sensor measures the vector field in all three spatial directions. In our study, experimentally measured maximum SAR value with the phantom in the MRI systems under the maximum RF irradiation was approximately 28.8 W/kg at 3 T. The numerical simulation showed that a highly non-uniform electric field was generated in the vicinity of the head and tail of the artificial hip joints, resulting in high SAR and RF-induced heating, in agreement with experimental measurements.

In the absence of the hip implants, local SAR levels in the body of the implants were slightly higher than those in the head and tail; however, in the case of the hip joints in place, local SAR values in the head and tail regions were much higher than those in the body part of the implants. Local SAR levels with the implants were at least five times higher at the head and seven times higher at the tail than those without the implants at the same measurement locations.

According to IEC standard 60601-2-33, the maximum SAR limit for the whole body is 2.0 W/kg, and the maximum temperature limit is 39 °C and the core temperature increase is limited to 0.5°C^6^. In our study, the temperature rises measured via the FBG sensors indicate that RF-induced heating appear in head and tail regions of the artificial hip joints. Furthermore, the measured peak temperature increase (= 4.22 °C) occurred in the tail region of the hip joint, and the measured high temperature rise (= 2.84 °C) was also shown at the head of the implant at 1.5 T. Similarly results hold at 3 T as well. The half wave length of the RF field in human tissue is about 13 cm at 3 T and 25 cm at 1.5 T. A conductor with a length of the half wave will be in resonance with the RF and induce high levels of heating. The total length of the hip joint is about 20 cm in this study (this will not be true if the cup and tail are insulated from each other), in the middle of the half wave length of 1.5 T and 3 T. Based on these considerations, the artificial hip is not expected to cause a very high temperature elevation, which is consistent with the result of this study. As can be seen from the measured temperature at the tip of the implant as a function of scan time, the measured temperature increased nonlinearly due to the reduction of heating effect of the hip implant by heat transfer to the surrounding gelled-saline phantom and the eddy current variation at the tip of the implant with a large curvature.

In this study, a single scan time ranged from 3 to 12 min. The ASTM F2182-09 standard test method requires 15 min of continuous RF irradiation because it is considered to be the reasonable maximum time for a single clinical scan and the temperature tends to increase almost proportionality to the RF irradiation time^8^. In reality, most clinical MRI examinations require up to one hour of total scan time depending on the body part being imaged, clinical indications and patient tolerance. Although each individual sequence usually does not exceed 15 min, the pauses between the sequences can be very short, and the accumulated heating could exceed that from one 15-min continuous scan.

Compared with the previous studies, the use of E-field sensor allows direct and reliable measurement of the RF electric field and SAR level, and it also allows us to compare SAR levels at different locations inside the gelled-saline phantom. A large variability of SAR was observed. High SAR levels measured by the E-field sensors were found at the head and tail of the artificial hip joints. The measured local SAR values are analogous to the numerical simulation results and also exceeded the allowed limits. The positions indicating temperature rises in the titanium alloy implant in our study were consistent with each other in the numerical calculations and the experimental measurements at two different magnetic field strengths of 1.5 T and 3 T with three different MRI sequences. High temperature rises were observed near not only the tail but also the head parts of the hip implants. One other important feature of our study is the calorimetric study using 28 temperature sensors for each artificial hip joint (a total of 56 temperature sensors), compared with only four sensors commonly used in the ASTM F2182-09 standard. Using a large number of sensors allows more accurate, reliable and realistic assessment of the temperature distribution without missing the hottest areas and SAR values obtained from the E-field measurement and the temperature measurement also agree well with each other.

There are some limitations of this study. First, the estimated and measured local fields may not occur in vivo due to differences in material properties and geometries. Second, blood perfusion substantially decreases the temperature rise during clinical imaging. Third, the temperature rises, but it may or may not cause substantial damage. Fourth, the probability of significant tissue damage occurring in an asymptomatic fashion may be exceptionally low. Last, we should also consider the surgical practice of using overheated cement when placing total hip replacement. This certainly results in a much higher local temperature rise than was estimated in this study. This study provides an opportunity to show discrepancies between experimental-based safety concerns (measured in simulated tissue environments) and actual clinical practices (with decades of safe scans of hip replacement)^36^.

In addition, it would be recommended to consider not only physical-based safety-related findings but also the overall impact on the clinical outcome in patients who do not receive proper image evaluation. This can result in over-emphasizing speculative safety issues or poor utilization of useful diagnostic imaging tools. The results presented in this study are not intended to be generalized beyond this configuration without additional data. The results should not be inferred from a more general or less controlled imaging situation without supporting data indicating safety.

In conclusion, results and experimental methods obtained from this study can play an important role in evaluating patient safety in acquiring medical images from patients with artificial hip joints implanted into the body in a magnetic resonance environment. In addition, our methods are applicable to other artificial hip joints, other MRI vendors or other scan sequences to estimate RF heating and body tissue injuries by measuring the SAR and temperature rise.

## Methods

### MRI systems

SAR values were measured on five MRI scanners: a 1.5 T Achieva (Philips Healthcare), a 1.5 T Signa Excite (GE Healthcare), a 3.0 T Magnetom Verio (Siemens Healthcare), and two of the same model 3.0 T Achieva (Philips Healthcare) systems. Three different MRI sequences with various SAR values supplied by the MRI systems were used on the scanners. Image acquisition parameters are listed in Supplementary Table 1.

The RF excitation power was transmitted by an integrated RF body coil (multi-transmit mode = “NO” at 3.0 T Philips). A four- or eight-channel body array coil was used as a receive coil in the present study. Without experiments on humans or the use of human tissue samples, we performed phantom experiments mimicking the human body.

The whole-body SAR is the total power absorbed by the whole body divided by the weight of the whole body^37^. The “effective weight” of the phantom was the weight of a whole human body that would absorb the same amount of RF power as our 21 L of gelled saline phantom. The effective weight of 21 kg (= 33% of the weight of an equivalent sized complete Korean adults) was entered into the MRI system at registration.

### Phantom morphology

A human lower body-shaped phantom (Supplementary Fig. 3) was constructed on the basis of Korean adult anthropometric reference data for 6413 Korean subjects (3192 males and 3221 females, 16 to 69 years of age) (Supplementary Table 2)^38^.

The air-tight plastic phantom container (15 mm thickness) was filled with a volume of approximately 20.8 L of a hydroxy-ethyl cellulose (HEC) gelled-saline solution consisting of 24.2 g of NaCl, 483.6 g of HEC powder and 20.8 L of distilled water, simulating human tissue, as described in the ASTM International standard method for SAR measurements^8^.

The gel thermal properties (thermal diffusivity = 1.4 × 10^−7^ m^2^/s and heat capacity = 4156 J/(kg °C)) were measured with a thermal property analyzer (KD2, Decagon Devices Inc., Pullman, WA, USA). The electric conductivity (σ = 0.48 ± 0.04 S/m at 64 MHz and 0.49 ± 0.04 S/m at 128 MHz) and relative electric permittivity (ε_r_ = 76.48 ± 3.98 at 64 MHz and 76.22 ± 4.12 at 128 MHz) of the gel solution were measured using a dielectric assessment kit (DAK-12, SPEAG Ltd., Zurich, Switzerland). The density (*ρ* = 1.0095 ± 0.0008 g/cm^3^ at 22.4 °C) was measured by a specific gravity cup (pycnometer, SHEEN 1501/50, TQC Sheen Ltd., United Kingdom).

### An artificial hip joint

**Figure 4:**
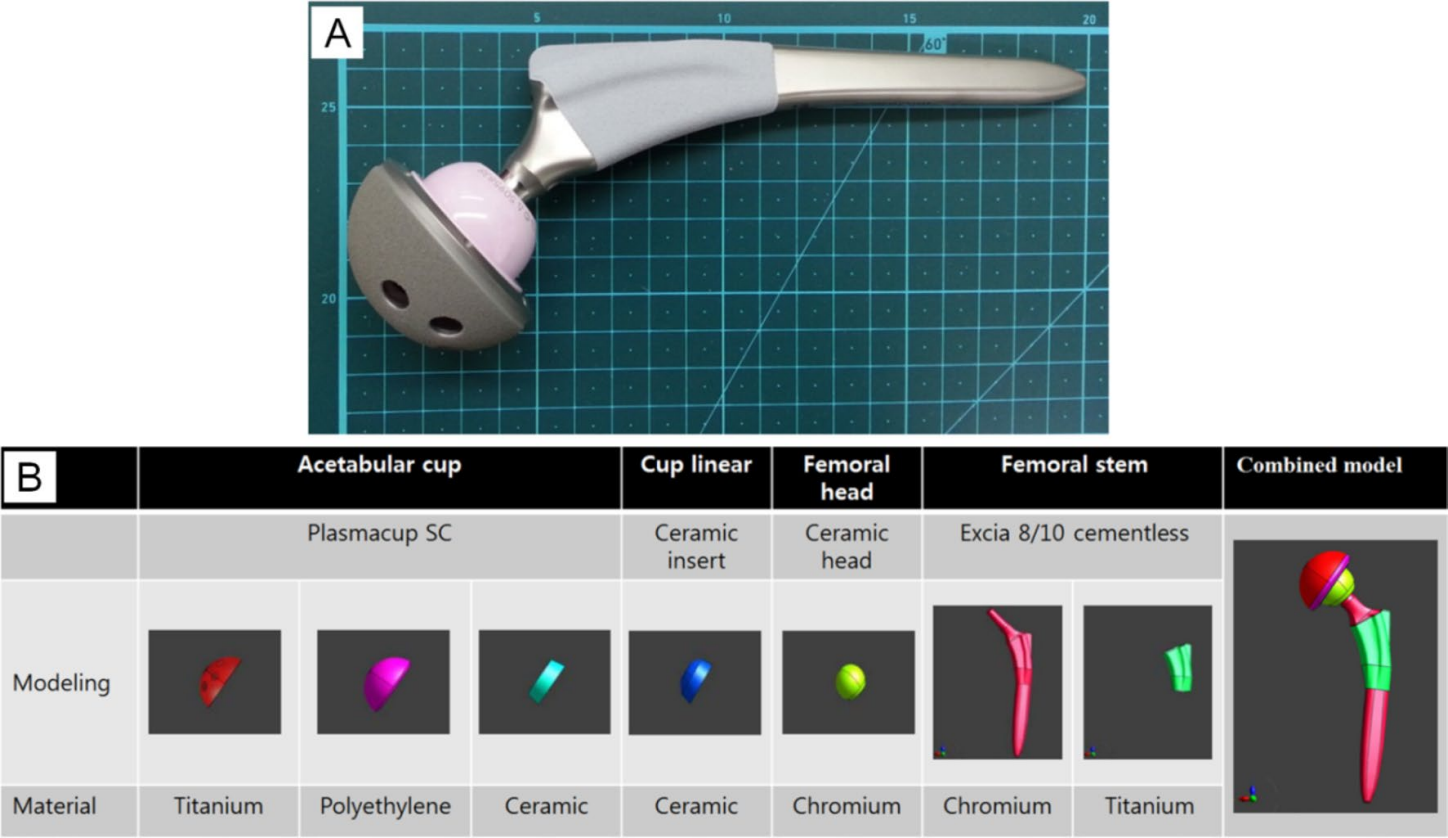
An artificial hip joint (**A**) and modeling for the artificial hip joint (**B**) using AutoCAD 2015 software (Autodesk, San Rafael, USA. https://www.autodesk.com/). The head and tail of the implant are connected electronically.

**Figure 5:**
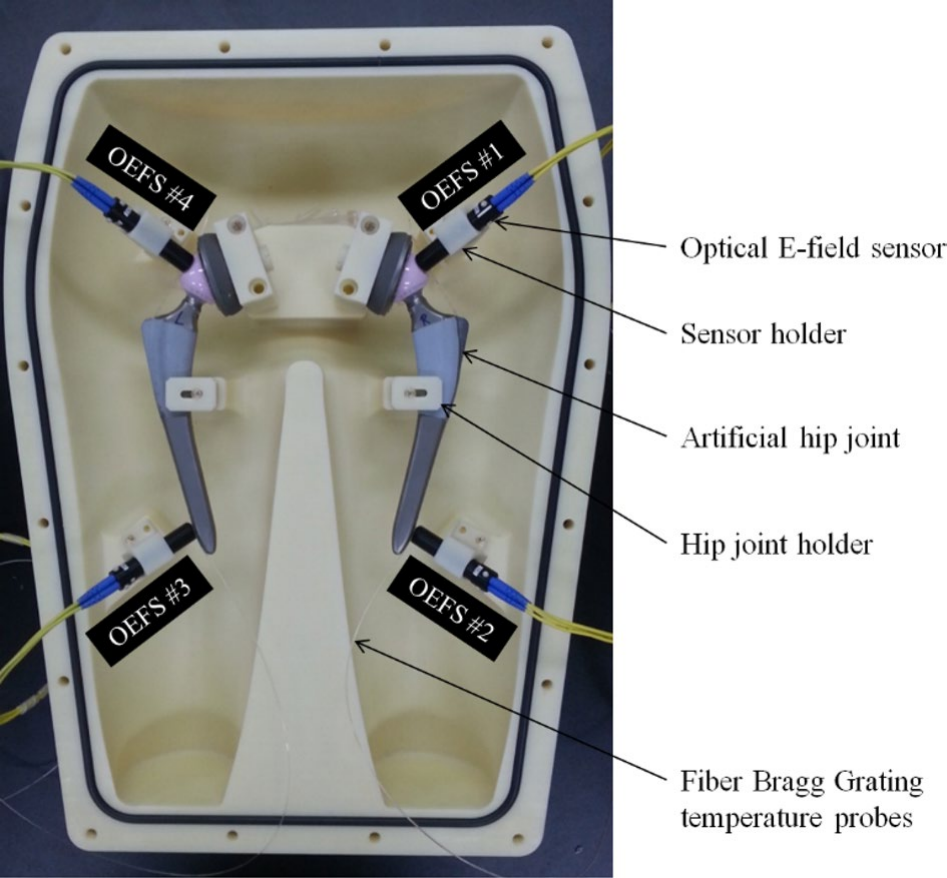
Locations of four optical E-field sensors (OEFS) and two optic-fiber Bragg grating (FBG) temperature sensor arrays on the surface of the artificial hip joints.

A hip replacement implant consists of an acetabular cup (B. Braun Medical Inc., Plasmacup SC, Model No. NH050T), a cup liner (Exactech Inc., Biolox delta, ceramic inserts, Model No. NH050T), a femoral head (Exactech Inc., Biolox delta, ceramic femoral head, Model No. NJ106D) and a femoral stem (B. Braun Medical Inc., Excia 8/10 cementless, Model No. NC413T) (Fig. 4A). The length unit is centimeter. The artificial hip joint was immersed inside the human lower body-shaped phantom filled with the HEC gelled-saline solution as shown schematically in Fig. 5.

### Numerical simulation of SAR and RF-induced heating at 3 T

The commercially available Sim4Life version 4.4 software (Zurich Med Tech, Zurich, Switzerland. https://zmt.swiss/sim4life/) was used to calculate the SAR distribution with the electromagnetics full wave solver (P-EM-FDTD) and temperature distribution with the thermodynamics solver (P-THERMAL) in the phantom model. A high-pass birdcage coil is commonly used as a body coil for homogeneous excitation^39^. The high-pass birdcage body coil (62 cm diameter and 65 cm length) was modeled with 8 copper rungs attached to a copper shield (70 cm diameter and 120 cm length) via 28 copper strips (2.5 cm × 2.2 cm). Each rung consists of three separate copper strips, each of length 20 cm and width 2.5 cm, with four capacitor junctions. The dimensions of the coil and shield were based on those of the typical body coil. The body coil was tuned unloaded. In terms of a 4-port drive modeling, four voltage sources were inserted into gaps at the rear end-ring (superior end of head-first body model) at the 45°, 135°, 225° and 315° angular positions. The voltage sources had the same voltage amplitudes and the voltage phase values were 0°, 90°, 180° and 270°^39^. When tuning the body coil in Sim4Life, point |B_1_^+^| sensors were placed in several locations including the middle of the coil, and Gaussian pulses were fed to the voltage sources. By observing the spectral peaks of the |B_1_^+^| sensors, the capacitor values were adjusted until the desired homogenous model frequency shifts to 127.88 MHz. Knowing both SAR distribution and the location of high SAR around the artificial hip joints was of primary interest in numerical simulation of SAR.

Modeling for the artificial hip joint was performed according to the sizes and materials of the hip joint components (Fig. 4B). The phantom was positioned with the head-first entry in the center of the coil rungs and meshed to a finite-difference time-domain (FDTD) cell size of 3 × 3 × 3 mm^3^. Uniaxial perfectly matched layer (UPML) was used as absorbing boundary conditions. The back of each model was located 18 cm away from the furthest rung in the posterior direction. The human tissue properties were represented by Gabriel parameters at 128 MHz, which were used in the dispersive fitting (DISPFIT) tool, selecting minimum and maximum number of dispersive poles = 1 and 5, respectively. Numerical FDTD simulation was performed on an Intel Xeon Hexa-core 2.3 GHz CPU with an Nvidia Tesla C2075 GPU.

The SAR distribution of the phantom is irradiated with RF power was calculated using the following equation^39^:

1 $$SAR\left(\overrightarrow{r},t,f\right)=\frac{\upsigma \left(f\right)\times {\left|\overrightarrow{E}\left(\overrightarrow{r},t,f\right)\right|}^{2}}{2\rho }$$

where *σ* is the electric conductivity of the tissue, *ρ* is the volumetric density, $\overrightarrow{E}$ is the three-component electric field strength vector (*x, y, z*), $\overrightarrow{r}$ is the three-component spatial location vector (*x, y, z*), *f* is the RF frequency and *t* is the time elapsed^8^. First, we measured directly the electric filed strengths in three orthogonal directions using the E-field probes and then obtained the amplitude of the electric field vector. Using Eq. (1), the SAR values were obtained without following the ASTM standard test method. When continuous wave RF power was applied, the steady-state solution of $\overrightarrow{E}$ was used directly to compute SAR. After each electromagnetic simulation with continuous wave sources, the SAR was set to 2.0 W/kg, which is the IEC 6 min whole-body SAR limit in the normal mode^6^. To check MR heating for the passive implants, we referred to ASTM F2181 standard method^8^. An RF magnetic field generating the whole-body averaged SAR of 2 W/kg over the phantom volume was applied to characterize the SAR and the temperature elevation. The SAR values were then normalized to the whole-body averaged SAR of 2 W/kg.

The temperature of the phantom was modeled with a finite difference implementation of the Pennes’ bioheat transfer equation^40^. For simplicity, the perfusion rate was assumed to be independent of time and temperature, with a constant temperature of 37 °C in the phantom and the ambient temperature of 25 °C.

### Independent SAR measurement using optical electric field sensors

Four optical E-field sensors (OEFS, Model: SH-10EL, SEIKOH GIKEN Inc., Norcross, USA) were located near the femoral heads and tails of the femoral stems showing the maximum SAR on the basis of on the simulation results (Fig. 5). The OEFS sensors were used to measure three-axis direction E-field strengths on each MRI scanner during repeated scans. The E-field sensors were calibrated with traceability using a transverse electromagnetic (TEM) cell. The SAR was determined using Eq. (1). The experimental setup for SAR measurement is shown in Supplementary Fig. 4. The optical E-field sensors were connected with a controller (Model: C3-0355, SEIKOH GIKEN Inc., Norcross, USA), which was connected with a spectrum analyzer (Model: 8560E, Agilent Technologies Inc., Santa Clara, CA, USA). The spectrum analyzer was connected with a personal computer using a general purpose interface bus (GP-IB) to read and record three-dimensional E-field strengths around the artificial hip joints. For the measurement conditions of the controller and the spectrum analyzer, the center frequency was set to 64 MHz at 1.5 T and 128 MHz at 3 T with span =  ± 0.5 MHz; sweep set to continuous; the reference level and the display scale (LOG) of the amplitude set to -10 dBm and 10 dB; Resolution bandwidth (RBW) set to 0.005 MHz and video bandwidth (VBW) set to 0.001 MHz; the timeout response set to 1 s; and the channel change interval at the time of automatic measurement set to 100 ms with sampling count = 200.

### Temperature measurements

Four fiber-optic temperature sensors (Neoptix Inc., Quebec, Canada) were placed at the periphery of the gelled-saline filled phantom with the presence of the artificial hip joints to certify that there was minimal heat loss to the environment during the measurements similar to the method as described previously^15^. We measured the initial temperatures of the sensors positioned inside the phantom and the time it took to reach equilibrium with the environment. We considered that thermal equilibrium has been reached when the difference between temperatures measured by the temperature sensors in the phantom and temperature inside the magnet bore was less than 0.05 °C.

**Figure 6:**
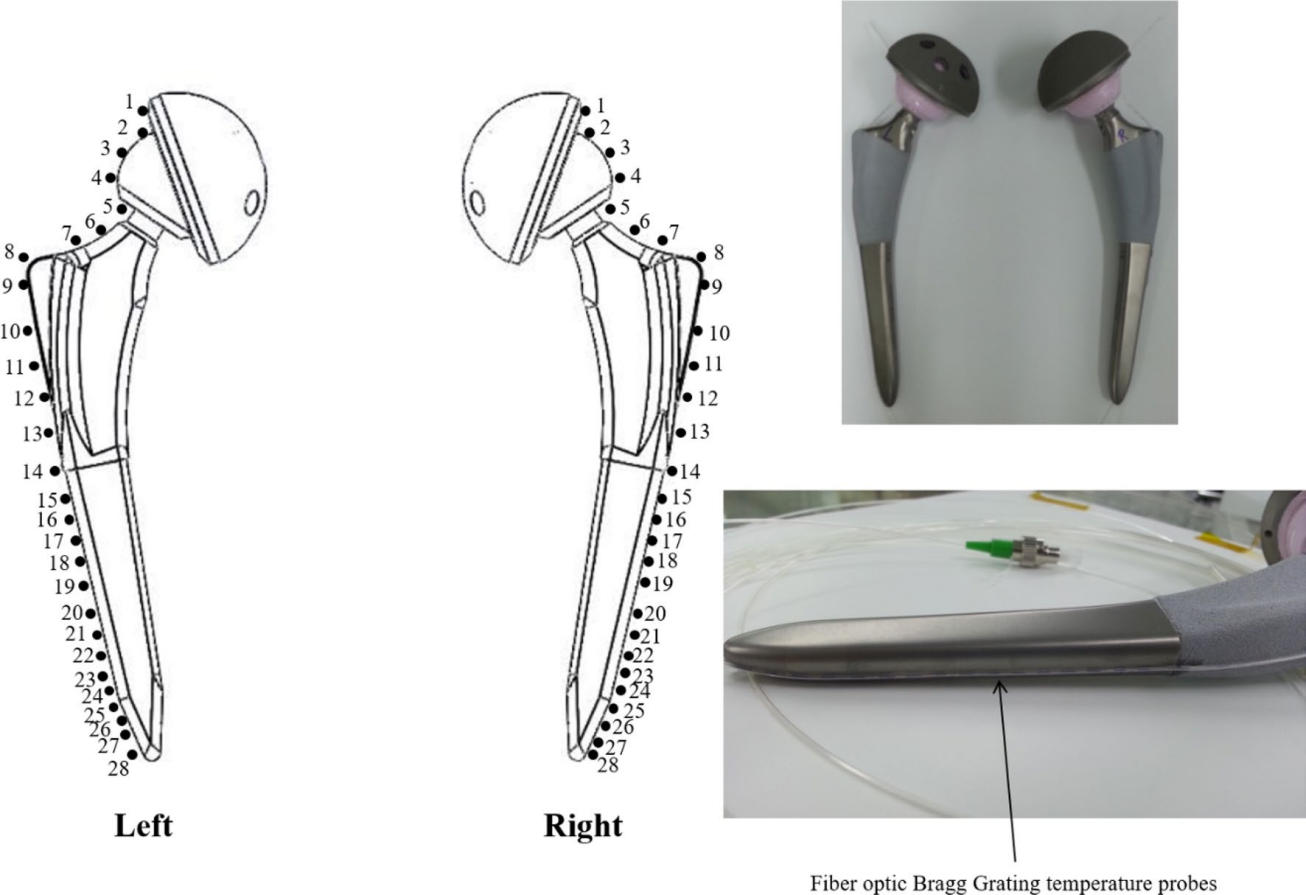
Schematic diagram and photographs showing the locations of fiber-optic Bragg grating (FBG) temperature sensors around artificial hip joints.

Fifty-six fiber Bragg grating (FBG, SJ Photonics Co., South Korea, wavelength range from 1510 to1590 nm) temperature sensors (28 FBG sensors on each joint) were located on a couple of the artificial hip joints to measure the temperature variations of the artificial hip joints due to RF-induced heating (Fig. 6). The FBG temperature sensors were connected with an interrogator (Model: SJP-M-02-950, SJ Photonics Co. Ltd., South Korea), which was connected with the personal computer to read and record the wavelength with a frequency of 970 Hz around the hip joints during MRI scanning. Finally, the measured wavelength was converted to temperature.

These FBG-based temperature sensors (0.3 mm diameter) minimize perturbations of RF fields. The FBG temperature system has a temperature resolution of 0.01 °C. The FBG sensors were calibrated with traceability. On each MRI scanner, temperature before and after MRI scanning was recorded as the temperature varies, and the difference between the measured temperatures was obtained.

### Reference local SAR without the presence of hip joint implant and SAR with the implant

Similar to the ASTM standard test method^8^, local temperature changes were first measured without the hip implant at the same temperature sensor locations as previously described in the temperature measurements. Temperature changes were then measured with the presence of the implant at the same hot positions as measured previously. These measured temperature rises were used to determine the local SAR without the implant and SAR with the implant at each of the fifty-six temperature sensor locations in the gelled-saline phantom. All measurements were repeated three times. SAR obtained from this temperature measurement with the FBG-based temperature sensors was compared to those from the E-field measurement using the optical E-field sensors.

### Placement of artificial hip joints, hip joint holders, OEFS probes and FBG sensors

The artificial hip joints, hip joint holders, OEFS probes and FBG sensors were deployed and immersed inside the phantom filled with the HEC gelled-saline solution to prevent the local electric field and thermal environment from being disturbed according to the ASTM guideline^8^. A vacuum was created in the phantom container to eliminate air bubbles inside the gel mixture. The entire experiment setup was placed in the magnet bore of the MR scanner for at least 24 h to establish thermal equilibrium with the environment before MRI scanning.

## Supplementary information

Supplementary Information 1.

## Data Availability

Experimental data from this study are available to interested readers upon reasonable request.
