# Supplementary material for: Measurement and evaluation of specific absorption rate and temperature elevation caused by an artificial hip joint during MRI scanning
Source: Sci Rep. 2021 Jan 13;11:1134. doi: 10.1038/s41598-020-80828-7 (PMC7807097; doi:10.1038/s41598-020-80828-7)
Supplement: Supplementary file 1 — Supplementary Information 1. [file 41598_2020_80828_MOESM1_ESM.pdf]

Supplementary Material

Measurement and evaluation of specific absorption rate and temperature elevation caused by an artificial hip joint during MRI scanning

Youngseob Seo[1], Zhiyue J. Wang[2,3]

[1]Division of Chemical and Biological Metrology, Korea Research Institute of Standards and Science, Daejeon, Republic of Korea

[2]Department of Radiology, University of Texas Southwestern Medical Center, Dallas, Texas, USA

[3]Department of Radiology, Children's Health, Dallas, Texas, USA

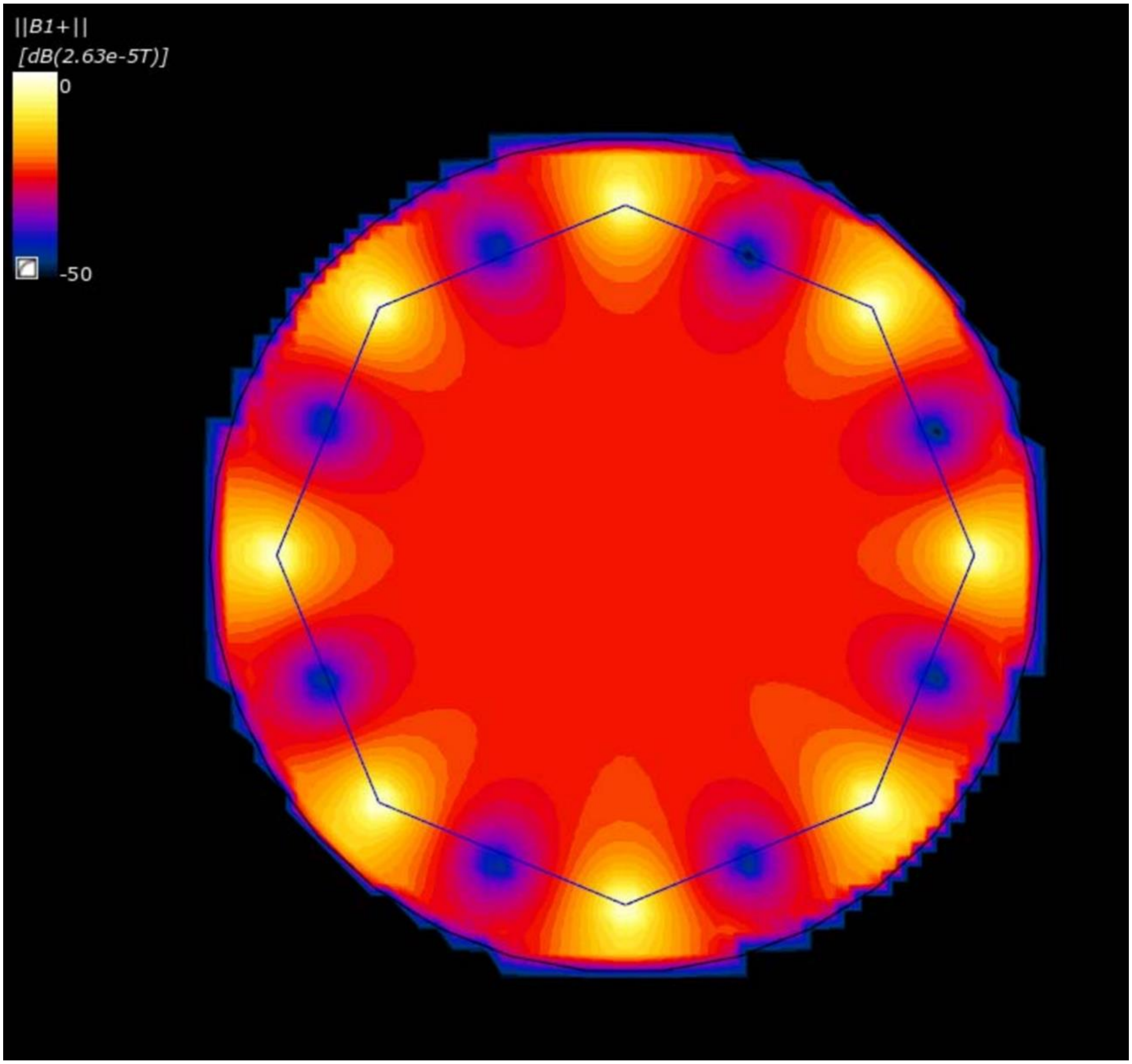

Supplementary Figure 1. RF magnetic field |B1+| of an axial slice in the unloaded high-pass birdcage RF body coil using Sim4Life version 4.4 software (Zurich Med Tech, Zurich, Switzerland. https://zmt.swiss/sim4life/) (normalized to the maximum value).

Supplementary Table 1. MRI sequences and image acquisition parameters at 1.5 T and 3.0 T. (Abbreviations: n/a, not applicable; NSA, No. of signal averages; TE, echo time; TI, inversion time; TIRM, turbo inversion recovery magnitude; TR, repetition time; TSE, turbo spin echo; SPIR, spectral pre-saturation with inversion recovery.)

	GE 1.5 T w/ 8 CH body upper coil			Philips 1.5 T w/ a torso coil		
	T1w TSE	T2w TSE	IR TSE	T1w TSE	T1w SPIR	T2w TSE
Scanner-reported whole body SAR [W/kg]	1.53	1.48	1.23	1.48	1.2	0.88
TR/TE [ms]	600/10.7	3500/96.2	4000/67.4	100/15	282/8.0	4003/110
TI [ms]	n/a	n/a	135	n/a	200	n/a
Field of view [mm^2]	450×450	450×450	450×450	450×460	450×450	450×450
No. of slices	13	13	24	30	12	20
Slice thickness [mm]	6	4	6	3	4	8
Acquisition matrix	256×256	256×256	256×256	564×458	452×360	452×328
Voxel size [mm^3]	1.76×1.76×6.0	1.76×1.76×6.0	1.76×1.76×6.0	0.8×1.0×3.0	1.0×1.25×4.0	1.0×1.37×8.0
Slice orientation	Coronal	Coronal	Transversal	Coronal	Transversal	Coronal
Phase-encoding direction	Head-Foot	Head-Foot	Head-Foot	Head-Foot	Anterior-Posterior	Head-Foot
NSA	1	1	1	2	2	1
Scan time	3 min 32 s	5 min 20 s	5 min 20 s	12 min 12 s	6 min	3 min 30 s

Bandwidth [Hz/pixel]	195.3	244.1	287.9	292.7	291.1	335.2

	Siemens 3 T w/ Body MATRIX a Tim coil			Philips 3 T w/ SENSE XL torso		
	T1w TSE	T1 TIRM	T2w TSE	T1w TSE	T1w SPIR	T2w TSE
Scanner-reported whole body SAR [W/kg]	1.1	1.5	1.1	1.5	1.5	1.5
TR/TE [ms]	800/10	1150/9.3	4710/110	100/15	282/8.0	4003/110
TI [ms]	n/a	220	n/a	n/a	200	n/a
Field of view [mm^2]	450×450	450×450	450×450	450×460	450×450	450×450
No. of slices	10	8	10	30	12	20
Slice thickness [mm]	6	6	6	3	4	8
Acquisition matrix	400×400	256×256	200×154 (reconstructed to 400×400)	564×458	452×360	452×328
Voxel size [mm^3]	1.13×1.13×6	1.76×1.76×6	1.13×1.13×6	0.8×1.0×3.0	1.0×1.25×4.0	1.0×1.37×8.0
Slice orientation	Coronal	Coronal	Coronal	Coronal	Transversal	Coronal

Phase-encoding direction	Head-Foot	Head-Foot	Head-Foot	Head-Foot	Anterior-Posterior	Head-Foot
NSA	2	1	4	2	2	1
Scan time	4 min 33 s	4 min 44 s	4 min 26 s	5 min 6 s	6 min 8 s	3 min 40 s
Bandwidth [Hz/pixel]	290	260	334	292.7	291.1	335.2

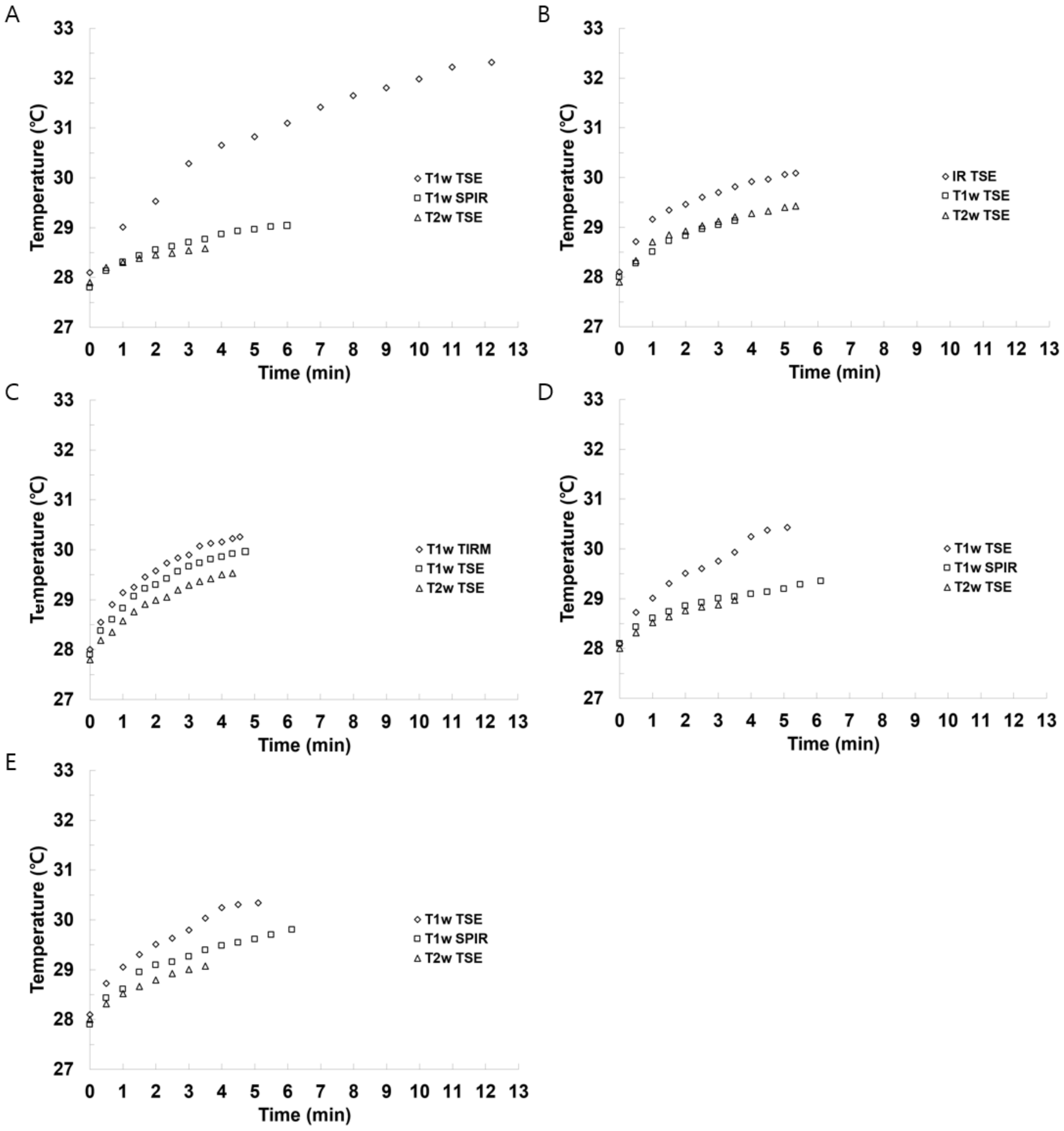

Supplementary Figure 2. Experimentally measured temperature as a function of scan time via fiber-optic Bragg grating temperature sensors at one tip location on the left side of artificial hip implants for three different sequences at 1.5 T Philip (A), 1.5 T GE (B), 3 T Siemens (C) and two same model 3 T Philips (D and E). The location of the temperature sensor was No. 25 as described in Figure 6. Temporal temperature increased gradually with continuous RF irradiation during MRI scans.

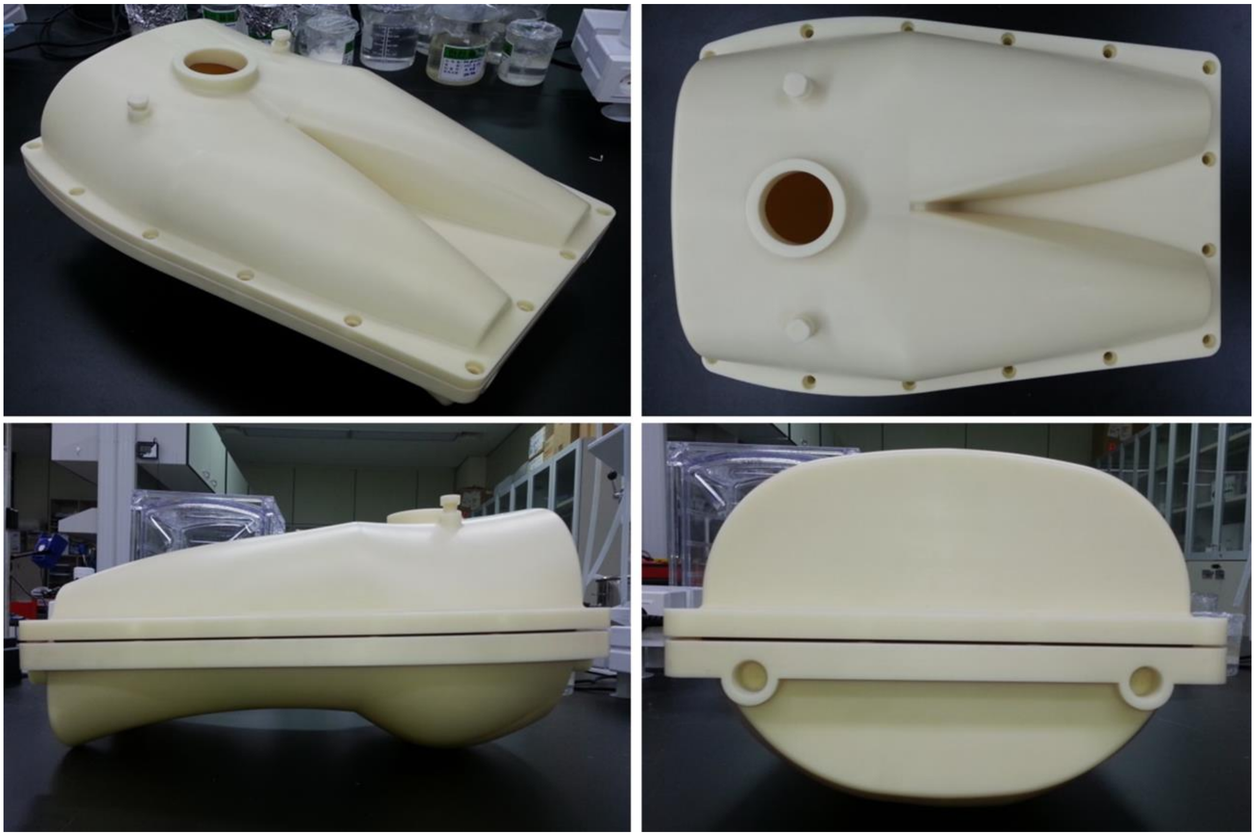

Supplementary Figure 3. Phantom morphology mimics the shape of the human lower body from waist to knee as described in Supplementary Table 2.

Supplementary Table 2. Korean reference values for the anthropometric parameters (16 to 69 years of age)

**Anthropometric parameters**	**Male**	**Female**
Waist circumference (omphalion) (cm)	86.5	80.6
Hip circumference (cm)	96.3	93.4
Waist breadth (natural indentation) (cm)	28.9	26.5
Hip width (cm)	33.0	32.7
Waist depth (natural indentation) (cm)	22.0	19.1
Hip depth (cm)	22.7	21.9
Thigh circumference (cm)	57.5	55.2
Mid-thigh circumference (cm)	52.6	49.7
Knee circumference (cm)	37.2	35.2
Thigh vertical length (cm)	29.6	27.7
Waist to hip length (cm)	20.7	19.5
Weight (kg)	74	57

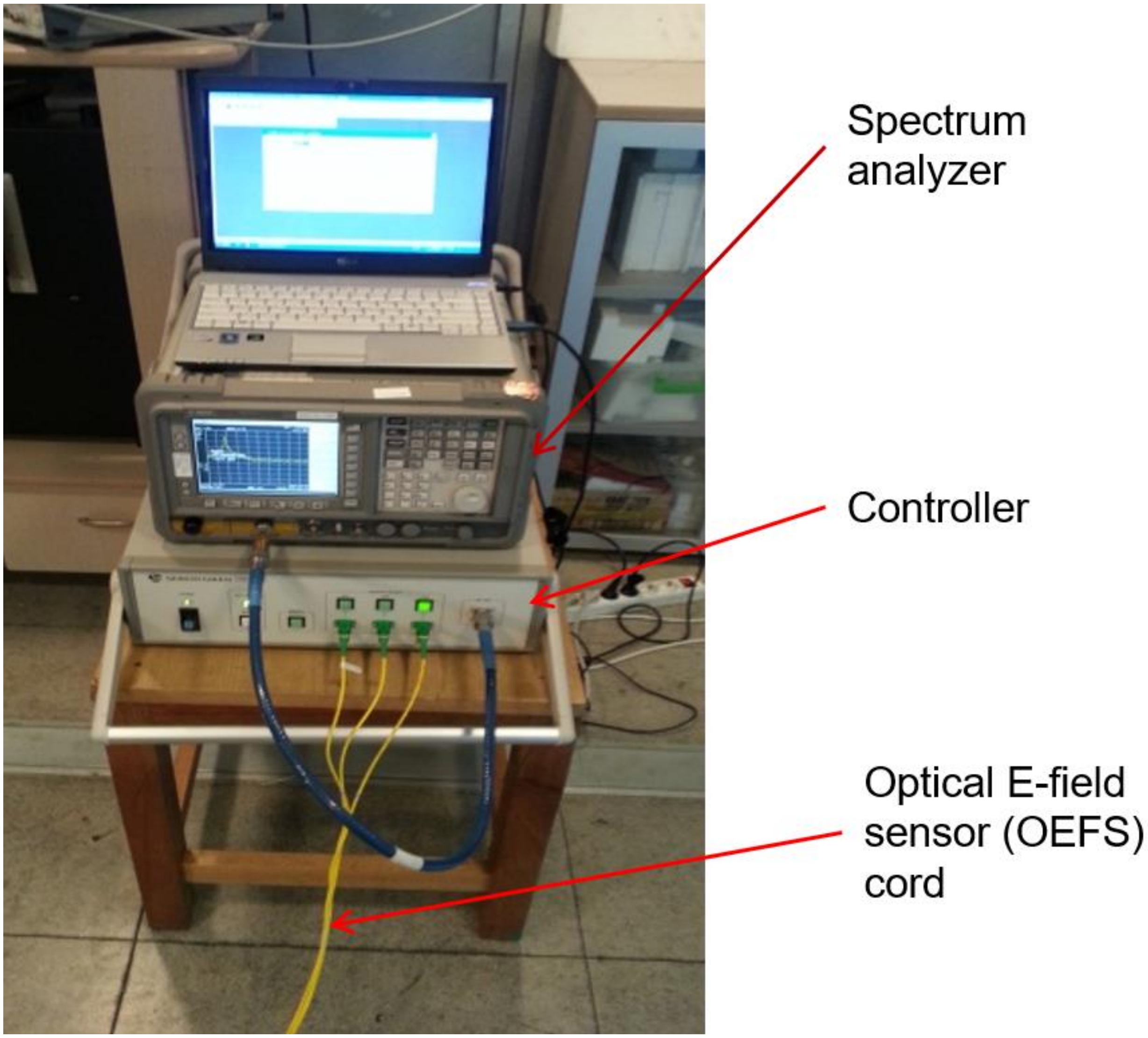

Supplementary Figure 4. Overview of the experimental setup for independent measurement of electric field strength inside the phantom during MRI scanning.
